# Bullying victimization among preadolescents in a community-based sample in Canada: a latent class analysis

**DOI:** 10.1186/s13104-020-04989-4

**Published:** 2020-03-07

**Authors:** Adiba Ashrafi, Cindy Xin Feng, Cory Neudorf, Khrisha B. Alphonsus

**Affiliations:** 1 Mailman School of Public Health, Columbia University, New York, USA; 2 School of Epidemiology and Public Health, Faculty of Medicine, University of Ottawa, 600 Peter Morand Crescent, Ottawa, ON K1G 5Z3 Canada; 3 Health Surveillance & Reporting, Saskatchewan Health Authority, Community Health and Epidemiology, University of Saskatchewan, Saskatoon, SK Canada; 4 School of Public Health, University of Saskatchewan, Saskatoon, Canada

**Keywords:** Latent class analyses, Bullying victimization, Suicidal ideation, Preadolescence, School violence

## Abstract

**Objective:**

Bullying victimization among adolescents has been well-recognized as a behavior associated with adverse psychological and mental health outcomes. Most studies on bullying victimization have focused on adolescents, but research is sparse regarding school victimization among preadolescents before they transition to adolescence. This study sought to identify latent classes of different types of co-occurring bullying victimization, based on a sample of 3829 school students in grades 5–8, ages 9–14 in the year of 2011 from the Saskatoon Health Region, Saskatchewan, Canada.

**Results:**

Using a latent class analysis approach, the results uncovered three groups of victimized students, including those who were aggressively victimized (7.2%), moderately victimized (34.6%) and non-victimized (58.2%). Younger age and being overweight was associated with a higher likelihood of bullying victimization. Moderately and aggressively victimized students had greater probabilities of feeling like an outsider, experiencing anxiety, depressed moods, engaging in suicidal ideation and drinking when compared to non-victimized students. Peer and parent supports had significant protective effects against being victimized. Given the negative consequences of recurrent victimization among the preadolescents, it is imperative to address bullying incidents as they occur to prevent repeated transgressions, especially for those who suffer from multiple types of victimization.

## Introduction

Early adolescence, characterized as a phase of rapid physical and emotional growth, proves to be a difficult stage in life for children, as they are put at increased risk for bullying [1–3]. Bullying can be defined as inflicting intentional and repeated harm on someone when there is a real or perceived power imbalance [4]. There is growing evidence that bullying victimization among youths may have numerous negative mental health consequences, including psychiatric problems, substance use, delinquency, and aggression [5–9].

There are different types of peer victimization such as a physical attack, verbal harassment, social exclusion, spreading rumors, and cyberbullying and students may suffer from multiple types of victimization simultaneously. Latent class analysis (LCA) has been often used to uncover the latent co-occurring patterns of various types of bullying victimizations while accounting for the measurement errors [10, 11]. Understanding the nature of the latent classes of bullying victimization is essential to help schools develop prevention and intervention strategies for reducing bullying victimization. Previous research using LCA was often based on dichotomized responses of bullying occurrence; however, the frequency of repeated victimization reflects the severity of the bullying victimization, which should also be considered. Moreover, most studies using LCA to date have been conducted with children older than 10 years of age and middle school students [10, 11]. Research that focuses on a slightly younger age group is still relatively scarce.

To address the abovementioned gaps in the literature, the first purpose of our study was to uncover the latent classes of bullying victimization based on the self-reported bullying frequency according to the types of bullying victimizations (physical, verbal, social exclusion, and cyberbullying) through a series of LCA models among a community-based sample of preadolescents aged 9-14. The second purpose of this study is to identify factors associated with latent class membership.

## Main text

### Methods

A cross-sectional study was conducted among students, attending grades 5 to 8, ages 9–14 years based on the student health survey from the Saskatoon Health Region in Saskatchewan, Canada. A total of 5783 students (46.7%) responded. The final study sample included 3829 students, who completed the questions of interest related to bullying victimizations, socio-demographics, behavior, psychological, and relationship variables. Flowchart of exclusion criteria to arrive at the final analytic sample was reported in Figure S1 in Additional file 1. Four different types of bullying victimizations were measured: physically (i.e. hitting, pinching, kicking), verbally (i.e. spreading rumors, gossiping, insulting and teasing), socially (i.e. isolating and excluding others from the group) and electronically (i.e. threaten through the Internet, e-mail, phone or cellular phone text messages) in the past 4 weeks. There are four potential responses: never in 4 weeks, once or twice in 4 weeks, every week, or many times a week. Due to the small number of students who reported being bullied either every week or many times a week, these two categories were combined.

Previous studies have applied socio-ecological systems theory [12] to understand the determinants of bullying involvement, which are multi-level contexts that include individual characteristics (i.e. sociodemographic characteristics, behavior, and mental health variables), microsystem (interpersonal relationships of individuals within immediate settings, i.e. parental-youth relationship or peer relationships) [10]. In this study, individual-level factors include socio-demographic variables (gender, grade, school location, duration of stay in Canada, Aboriginal status, father’s employment status, mother’s employment status, living situation, and the number of schools attended in the past year), body weight (overweight/normal or underweight) based on self-reported weight and height, behavior variables (smoking and drinking behaviors where “never” individuals were compared to those who had “tried” the particular activity) and psychological variables including suicide ideation in the past year, feeling like an outsider, depressed mood (12-question scale shortened from the Centre for Epidemiological Studies Depression Scale (CES-D), ranging from 0 to 36), anxiety (eight-question scale ranging from 0 to 28), and self-esteem (five-question scale ranging from 0 to 20). Microsystem factors include the quality of relationships with parents and friends, which were categorized into poor, moderate, and good.

LCA was used to distinguish groups of students by bullying frequency according to different types of bullying [11, 13]. Bayesian information criterion (BIC) was measured to evaluate model fit, and the Vuong-Lo-Mendell-Rubin and the adjusted Lo-Mendell-Rubin likelihood ratio tests were used to compare between models of varying latent class size. Significant p-values in these tests suggest that the estimated model fits the data better than a model with one fewer group. LCA was run using the statistical software Mplus 7 [14]. Following LCA, multivariable multinomial regression of the latent class memberships was conducted to identify their important determinants using SPSS 24.0. Only significant explanatory variables at the 5% level of significance were included in the final model.

### Results

Using LCA, 2-, 3-, and 4-class models were considered to identify the latent classes of study participants by the bullying frequency according to different types of bullying victimization (Table 1). The 3-class model had the smallest BIC, making it the preferred model. The Vuong-Lo-Mendell-Rubin and the Adjusted Lo-Mendell-Rubin tests demonstrated that the model fit improved as additional groups were added from the 2-class model through to the 4-class model. However, the 4-class model (p < 0.05) was not as statistically significant as the 3-class model (p < 0.0001).

**Table 1:** Latent class analysis fit indices

Model selection criteria	2-Class	3-Class	4-Class
BIC	26,628.755	25,967.137	25,994.400
VUONG-LO-MENDELL-RUBIN LRT	p < 0.0001	p < 0.0001	p = 0.0158
LO-MENDELL-RUBIN ADJUSTED LRT	p < 0.0001	p < 0.0001	p = 0.0166

*BIC* Bayesian information criterion, *LRT* likelihood ratio test

Altogether, the 3-class model was chosen including latent classes: (1) non-victimized, (2) moderately victimized, and (3) aggressively victimized (Table **2**). The probability of students belonging to each group was 58.2%, 34.6%, and 7.2%, respectively. Among non-victimized children, the likelihood of never being bullied physically was 96.5%, verbally was 90.1%, socially was 96.9%, and electronically was 98.9%. In contrast, for the moderately victimized group, the probability of being bullied once or twice physically was 35.6%, verbally was 65.8%, socially was 53.1%, and electronically was 15.5%. Among aggressively victimized children, the probability of being frequently bullied physically was 30.1%, verbally was 80.6%, socially was 72.2%, and electronically was 19.1%. Additionally, the likelihood of these same children experiencing once or twice physical bullying, 44.5%, and occasional electronic bullying, 23.7%, was higher than the norm. The demographic characteristics by latent class membership are presented in Table S1 in Additional file 1.

**Table 2:** Item-response probabilities for latent classes of bullying frequencies according to different types of bullying victimization

Types of bullying	Bullying frequency	Overall average	Latent class *Item*-*response probabilities*		
			Non-victimized	Moderately victimized	Aggressively victimized
Physical	Never	0.791	0.965	0.628	0.254
	Once or twice	0.180	0.035	0.356	0.445
	Every week/many times a week	0.029	0.000	0.016	0.301
Verbal	Never	0.600	0.901	0.241	0.021
	Once or twice	0.301	0.099	0.658	0.173
	Every week/many times a week	0.099	0.000	0.100	0.806
Social	Never	0.713	0.969	0.439	0.085
	Once or twice	0.218	0.029	0.531	0.193
	Every week/many times a week	0.069	0.003	0.031	0.722
Electronic	Never	0.901	0.989	0.830	0.573
	Once or twice	0.079	0.011	0.155	0.237
	Every week/many times a week	0.020	0.000	0.015	0.191
*Latent class prevalence*			0.582	0.346	0.072

The results of evaluating the correlates of the class memberships based on multivariable multinomial regression (Table 3) indicated that preadolescents in grades 5–7 were more likely to be moderately and aggressively bullied than those in grade 8. Also, males were more likely to be aggressively victimized than females (OR: 1.79, CI 1.26–2.54), when compared to the non-victimized group, but this association was not evident among the moderately victimized group. In contrast, residency status, Aboriginal status, parents’ employment status, living situation, and changing schools were not significant explanatory variables of victimization, and thus, were excluded from the final model.

**Table 3:** Multivariable analysis for evaluating the correlates of the latent class memberships (n = 3829)

Covariates	Moderately victimized (n = 1326) vs. non-victimized (n = 2228)		Aggressively victimized (n = 275) vs. non-victimized (n = 2228)	
	Odds ratio (95% CI)	p-value	Odds ratio (95% CI)	p-value
Gender				
Male	0.98 (0.83–1.16)	0.815	1.79 (1.26–2.54)	0.001
Female	1.00	–	1.00	–
Grade				
Grade 5	2.06 (1.60–2.65)	< 0.001	3.85 (2.29–6.49)	< 0.001
Grade 6	1.83 (1.44–2.33)	< 0.001	3.04 (1.85–4.98)	< 0.001
Grade 7	1.81 (1.43–2.28)	< 0.001	2.58 (1.57–4.27)	< 0.001
Grade 8	1.00	–	1.00	–
Self-weight perception				
Overweight	1.48 (1.18–1.86)	0.001	2.31 (1.57–3.40)	< 0.001
Normal or Underweight	1.00	–	1.00	–
Suicide ideation in past year				
Yes	1.55 (1.00–2.40)	0.053	2.14 (1.18–3.87)	0.012
No	1.00	–	1.00	–
Feeling like an outsider	3.20 (2.82–3.63)	< 0.001	10.55 (8.37–13.29)	< 0.001
Depressed mood	1.02 (1.00–1.05)	0.105	1.10 (1.05–1.15)	< 0.001
Anxiety	1.09 (1.05–1.13)	< 0.001	1.13 (1.07–1.20)	< 0.001
Drinking				
Yes	1.91 (1.55–2.35)	< 0.001	2.87 (1.92–4.28)	< 0.001
No	1.00	–	1.00	–
Parent relationship	0.95 (0.93–0.97)	< 0.001	0.95 (0.91–0.99)	0.013
Friendships	0.89 (0.86–0.92)	< 0.001	0.87 (0.82–0.92)	< 0.001

Preadolescents with self-perception of being overweight were 1.48 (95% CI 1.18–1.86) and 2.31 (95% CI 1.57–3.40) times more likely to be moderately and aggressively victimized versus non-victimized, respectively. Aggressively victimized children were 2.14 (95% CI 1.18–3.87) times more likely than non-victimized to have suicide ideation. In both moderately victimized (OR: 3.20, 95% CI 2.82–3.63) and aggressively victimized groups (OR: 10.55, 95% CI 8.37–13.29) subgroups, preadolescents were more prone to feel like an outsider when compared to the non-victimized group. For every score increase in depressive symptom, the odds of being aggressively victimized was 1.10 (95% CI 1.05–1.15) times more likely than the odds of being non-victimized. Similarly, for every score increase in the level of reported anxiety symptoms, preadolescents were more likely to be moderately victimized (OR: 1.09, 95% 95% CI 1.05–1.13) and aggressively victimized (OR: 1.13, 95% CI 1.07–1.20) than non-victimized. Self-esteem was not a significant correlate of membership for any of the identified latent classes. For behavioral patterns, drinkers were more likely to be moderately victimized (OR: 1.91, 95% CI 1.55–2.35) and aggressively victimized (OR: 2.87, 95% CI 1.92–4.28) than non-victimized. Smoking was not associated with the identified latent class memberships.

A protective effect was observed among students who had better relationships with their parents. For every score increase in parent relationship, the odds of belonging to the moderately victimized class were 0.95 (95% CI 0.93–0.97) times and the odds of belonging to the aggressively victimized class were 0.95 (95% CI 0.91–0.99) times the odds of being non-victimized. The protective effect was greater for students who had many friends and who got along with their peers. For those with better friendships, the odds of being moderately and aggressively victimized were 0.89 (95% CI 0.86–0.92) times and 0.87 (95% CI 0.82–0.92) times the odds of being non-victimized, respectively.

### Discussion

The main strength of this study is that the LCA method was applied to bullying frequency according to different types of bullying rather than based on dichotomized bullying variables. The second strength is the current study focused on preadolescents before they transition to adolescence. Another strength is the use of community-based data containing rich information on a broad spectrum of determinants. Such data are rarely reported in the preadolescent literature.

Consistent with previous research, our findings further illustrate that students in younger grades were bullied more often versus students from older grades [1, 15, 16]. With the exception of students who display difficulties navigating this transitory period or who have social information-processing deficits, it is not surprising to observe greater victimization occurring in lower grades, where peer relations and peer group structure play a more salient role [1]. These results suggest the need for prevention and intervention efforts that might need to start as early as grade five. Our findings provide insight that being simultaneously bullied in multiple ways is common at this age. No significant gender and race differences were identified in association with latent class memberships.

This study provided further evidence of suicide ideation in the past year was significant among students who were aggressively victimized. Previous research found that risky suicidal behaviors were equally associated with non-physical bullying victims (e.g. verbal, social, cyber) as they were to physical bullying victims, despite extant research that contested relational aggression to be more causal of suicide ideation [17]. Besides suicide ideation, aggressively victimized students in this investigation were also more likely to display depressed mood symptomatology and report anxiety. Students who felt like an outsider at school had significantly higher chances of being either moderately or aggressively bullied, but especially the latter. This reaffirms findings from prior research, which suggest that victims exhibit tendencies to internalize their feelings [10] and that these feelings of not belonging combined with a poor bullying climate (e.g. perceived adult support) elevates the risk of being bullied often [18, 19].

Self-perception of being overweight is yet another factor that is more common in the moderately and aggressively victimized subgroups, which is consistent with present literature on discrimination towards individuals struggling with their body image and weight [20]. Weight loss preoccupation is common among bullies, victims, and bully-victims [4].

Drinking behavior was also found to be significantly associated with peer victimization, which may act as a coping mechanism for preadolescents who are bullied. Conversely, it is also possible that preadolescents who partake in underage drinking are more likely to be targeted for social exclusion, prejudice from their peers, etc.

Preadolescents, who had a stronger social network with peers and better relationships with their parents were less likely to be bullied because they could confide in their peers or parents when they faced challenges [21]. In turn, their peers could help them prevent and address bullying-related occurrences [2].

## Limitations

Due to the cross-sectional nature of this study, causal inferences cannot be drawn. Additionally, the questions on bullying type and frequency in the survey were asked for the past month; therefore, bullied earlier in the year or even bullied ever were not captured. Furthermore, perception of what constitutes bullying is highly variable from student to student, especially for verbal, social, and electronic bullying. A valid and reliable bullying assessment tool should be employed to obtain more accurate results [10, 13]. Finally, information on the contextual factors, such as including school and community characteristics are also very limited in this dataset.

## Supplementary information

**Additional file 1.** Additional figure and table.

## Data Availability

The data that support the findings of this study are available from Saskatoon Health Region Public Health Observatory, but restrictions apply to the availability of these data, which were used under license for the current study, and so are not publicly available. Data are however available from the Saskatoon Health Region Public Health Observatory upon reasonable request.

## Abbreviations

LCA: Latent class analysis
CES-D: Centre for epidemiological studies depression scale
BIC: Bayesian information criterion
