# Supplementary material for: Bullying victimization among preadolescents in a community-based sample in Canada: a latent class analysis
Source: BMC Res Notes. 2020 Mar 7;13:138. doi: 10.1186/s13104-020-04989-4 (PMC7060587; doi:10.1186/s13104-020-04989-4)
Supplement: Supplementary file 1 — Additional file 1. Additional figure and table. [file 13104_2020_4989_MOESM1_ESM.docx]

**Bullying victimization among preadolescents in a community-based sample in Canada: a latent class analysis**

**(Supplementary Materials)**


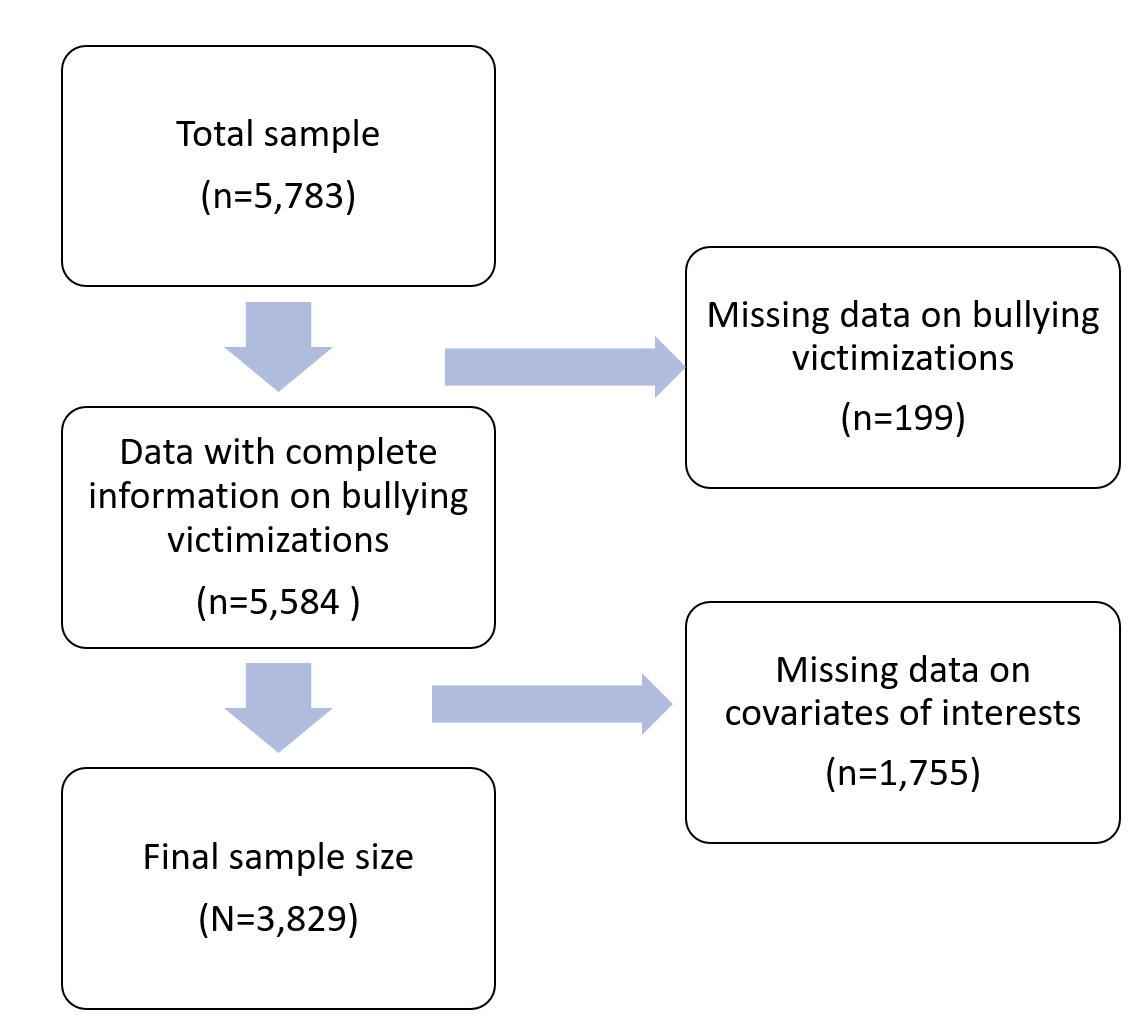


Figure S1: Flowchart of exclusion criteria to arrive at the final analytic sample

**TABLE S1.** Demographic Characteristics based on Latent Class Membership (n = 3,829).

|  | **Latent Class Membership Frequency (%)** | | |
| --- | --- | --- | --- |
|  | **Non-Victimized**  **(n = 2,228)** | **Verbally and socially occasionally Victimized**  **(n = 1,326)** | **Verbally and socially frequently**  **Victimized**  **(n = 275)** |
| **Gender** |  |  |  |
| Male | 1,124 (50.4%) | 569 (42.9%) | 129 (46.9%) |
| Female | 1,104 (49.6%) | 757 (57.1%) | 146 (53.1%) |
| **Grade** |  |  |  |
| Five | 461 (20.7%) | 290 (21.9%) | 63 (22.9%) |
| Six | 576 (25.9%) | 353 (26.6%) | 83 (30.2%) |
| Seven | 582 (26.1%) | 370 (27.9%) | 63 (22.9%) |
| Eight | 609 (27.3%) | 313 (23.6%) | 66 (24.0%) |
| **Duration of Stay in Canada** |  |  |  |
| Lifelong | 2,012 (90.3%) | 1,209 (91.2%) | 252 (91.6%) |
| Part of Life | 208 (9.3%) | 111 (8.4%) | 21 (7.6%) |
| **Aboriginal Status** |  |  |  |
| Yes | 175 (7.9%) | 140 (10.6%) | 42 (15.3%) |
| No | 1,993 (89.5%) | 1,153 (87.0%) | 226 (82.2%) |
| **Does your Father have a Job?** |  |  |  |
| Yes | 2,038 (91.5%) | 1,202 (90.6%) | 240 (87.3%) |
| No or Don’t know | 179 (8.0%) | 120 (9.0%) | 35 (12.7%) |
| **Does your Mother have a Job?** |  |  |  |
| Yes | 1,853 (83.2%) | 1,105 (83.3%) | 224 (81.5%) |
| No or Don’t know | 359 (16.1%) | 215 (16.2%) | 50 (18.2%) |
| **Who do you Live with Most of the Time?** | |  |  |
| Both parents | 1,762 (79.1%) | 971 (73.2%) | 185 (67.3%) |
| One parent or others (i.e. relative) | 454 (20.4%) | 350 (26.4%) | 88 (32.0%) |
| **Number of Schools Attended in the Past Year** | |  |  |
| One school | 2,066 (92.7%) | 1,208 (91.1%) | 244 (88.7%) |
| More than one school | 129 (5.8%) | 94 (7.1%) | 28 (10.2%) |

*Range of missing responses within latent classes for Duration of Stay in Canada = 0.4-0.7%, Aboriginal Status = 2.5-2.7%, Father Having a Job = 0.3-0.5%, Mother Having a Job = 0.4-0.7%, Living Situation with Parents = 0.4-0.7%, and Number of Schools Attended = 1.1-1.8%.
